# Comparative Performance of SARS-CoV-2 Detection Assays Using Seven Different Primer-Probe Sets and One Assay Kit

**DOI:** 10.1128/JCM.00557-20

**Published:** 2020-05-26

**Authors:** Arun K. Nalla, Amanda M. Casto, Meei-Li W. Huang, Garrett A. Perchetti, Reigran Sampoleo, Lasata Shrestha, Yulun Wei, Haiying Zhu, Keith R. Jerome, Alexander L. Greninger

**Affiliations:** a Department of Laboratory Medicine, University of Washington, Seattle, Washington, USA; b Department of Medicine, University of Washington, Seattle, Washington, USA; c Vaccine and Infectious Diseases Division, Fred Hutchinson Cancer Research Center, Seattle, Washington, USA; Boston Children's Hospital

**Keywords:** E-gene, N2, SARS-CoV-2, primer, probe

## Abstract

Nearly 400,000 people worldwide are known to have been infected with severe acute respiratory syndrome coronavirus 2 (SARS-CoV-2) beginning in December 2019. The virus has now spread to over 168 countries including the United States, where the first cluster of cases was observed in the Seattle metropolitan area in Washington. Given the rapid increase in the number of cases in many localities, the availability of accurate, high-throughput SARS-CoV-2 testing is vital to efforts to manage the current public health crisis. In the course of optimizing SARS-CoV-2 testing performed by the University of Washington Clinical Virology Lab (UW Virology Lab), we evaluated assays using seven different primer-probe sets and one assay kit. We found that the most sensitive assays were those that used the E-gene primer-probe set described by Corman et al. (V. M. Corman, O. Landt, M. Kaiser, R. Molenkamp, et al., Euro Surveill 25:2000045, 2020, https://doi.org/10.2807/1560-7917.ES.2020.25.3.2000045) and the N2 set developed by the CDC (Division of Viral Diseases, Centers for Disease Control and Prevention, 2020, https://www.cdc.gov/coronavirus/2019-ncov/downloads/rt-pcr-panel-primer-probes.pdf). All assays tested were found to be highly specific for SARS-CoV-2, with no cross-reactivity with other respiratory viruses observed in our analyses regardless of the primer-probe set or kit used. These results will provide valuable information to other clinical laboratories who are actively developing SARS-CoV-2 testing protocols at a time when increased testing capacity is urgently needed worldwide.

## Abstract (precis)

Nearly 400,000 people worldwide are known to have been infected with severe acute respiratory syndrome coronavirus 2 (SARS-CoV-2) beginning in December 2019. The virus has now spread to over 168 countries including the United States, where the first cluster of cases was observed in the Seattle metropolitan area in Washington. Given the rapid increase in the number of cases in many localities, the availability of accurate, high-throughput SARS-CoV-2 testing is vital to efforts to manage the current public health crisis.

## INTRODUCTION

In late December 2019, a cluster of cases of pneumonia of unclear etiology was first noted in Wuhan City in the Hubei Province of China (1). The etiology of these pneumonia cases, a novel type of coronavirus, was identified on 7 January 2020 (1). This novel coronavirus has now been named severe acute respiratory syndrome coronavirus 2 (SARS-CoV-2) while the disease it causes is known as coronavirus disease 2019 (COVID-19) (2).

Though the epidemic was originally concentrated in China, it has now spread around the world with approximately 380,000 known cases (see Note S1 in the supplemental material) as of 23 March 2020 (3). Cases outside China were observed early in the epidemic with the first detected in Thailand on 13 January 2020 (4). Soon afterward, cases were also identified in other East Asian countries including Japan and South Korea (1). The virus then spread to Europe and the Middle East, with the nations of Italy and Iran having particularly large numbers of identified cases per capita (3). Cases have now been identified, and deaths due to COVID-19 reported, on all 6 populated continents (3).

The Centers for Disease Control and Prevention (CDC) confirmed the first case of COVID-19 in the United States on 21 January. The infected person was a 35-year-old man who had recently returned to his home in Snohomish County, WA, after travelling to Wuhan City (5). No additional cases of COVID-19 were identified in Washington State until 28 February when two new cases were confirmed, one in Snohomish County and one in neighboring King County, where Seattle is located (6, 7). Since 28 February, the number of cases of COVID-19 in Washington has steadily increased and currently stands at 2,096 (3).

In response to the rapidly increasing number of confirmed and suspected cases of COVID-19 in the Seattle metropolitan area, the Clinical Virology Laboratory at the University of Washington (UW Virology Lab) began testing clinical specimens for SARS-CoV-2. Prior to and since making this testing service available, we have endeavored both to optimize the performance of our assay and to increase the rate at which we are able to test samples. We report here our observations comparing three different RNA extraction methods. We also compare the performances of SARS-CoV-2 detection assays using seven different primer-probe sets and one assay kit.

## MATERIALS AND METHODS

### Samples.

Three sets of samples were used in our analyses. First, we used a set of approximately 300 clinical respiratory samples sent to the UW Virology Lab for respiratory virus testing. These samples were in the form of nasopharyngeal or oropharyngeal swabs in viral transport medium and had not previously been tested for SARS-CoV-2. Second, we used a collection of nasal swabs in viral transport medium that are used to validate all assays performed by the UW Virology Lab. This collection includes samples positive for: rhinovirus (3 samples within the set), influenza B virus (2 samples), influenza A virus (2 samples), parainfluenza virus 1 (1 sample), parainfluenza virus 3 (2 samples), parainfluenza virus 4 (1 sample), adenovirus (2 samples), metapneumovirus (1 sample), bocavirus (2 samples), respiratory syncytial virus (2 samples), and coronavirus (25 samples). The coronaviruses included in the sample set are non-SARS-CoV-2 samples. Twenty-two negative samples are also included in this sample set. Finally, we obtained a set of 10 samples confirmed to be positive for SARS-CoV-2 by the Washington State Department of Health (WSDOH) Public Health Laboratories. These samples were also all nasopharyngeal or oropharyngeal swabs immersed in viral transport medium.

### RNA extraction.

RNA extraction from samples was performed using two different systems, the MagNA Pure LC 2.0 and the MagNA Pure 96 (Roche Life Sciences). For both systems, RNA extraction was performed according to the manufacturer’s instructions. For the MagNA Pure LC 2.0, 200 μl of each sample was subjected to extraction with an elution volume of 200 μl. For the MagNA Pure 96, 200 μl of each sample was subjected to extraction with an elution volume of either 50 or 100 μl. Five microliters of RNA in elution buffer was used in each SARS-CoV-2 detection assay.

### SARS-CoV-2 detection assays.

We used a total of 7 different primer-probe sets in our SARS-CoV-2 detection assays (see Table S1 in the supplemental material). The University of Washington (UW) RdRp primer-probe set was designed by the UW Virology Lab. Three additional primer-probe sets were designed as described in the work of Corman et al. (8); these will be referred to as the Corman N-gene, RdRp, and E-gene primer-probe sets. The Centers for Disease Control and Prevention (CDC) N1, N2, and N3 sets were developed by the CDC and have been published on the CDC website (9).

For all of the above primer-probe sets, real-time RT-PCR assays were performed using the AgPath-ID One Step RT-PCR kit (Life Technologies). Twenty-five microliters of reaction mix consists of 2× RT-PCR buffer, 25× enzyme mix, primers-probes, and 5 μl of extracted nucleic acid. Primer-probe concentrations were as recommended in the work of Corman et al. (8) and by a CDC-recommended protocol (9). RT-PCR was performed on an ABI 7500 real-time PCR system (Applied Biosystems) with the following cycle parameters: 10 min at 48°C for reverse transcription and 10 min of inactivation at 95°C followed by 40 cycles of 15 s at 95°C and 45 s at 60°C.

We also tested samples for SARS-CoV-2 using the BGI RT-PCR detection kit (BGI). These assays were conducted according to the kit manufacturer’s instructions.

A negative (human specimen control) was included in every RNA extraction procedure, and a nontemplate (water) control was included in every RT-PCR run. An internal control amplification, either RNase P or EXO (10), was performed to monitor RNA extraction and RT-PCR quality.

### Determination of SARS-CoV-2 sample copy number.

Dilutions of one SARS-CoV-2-positive clinical sample (SC5688) were used to evaluate the sensitivities of SARS-CoV-2 detection assays. To determine the viral copy number in SC5688, we obtained SARS-CoV-2 whole genomic RNA from the World Reference Center for Emerging Viruses and Arboviruses (WRCEVA) in Galveston, TX, which came at a reported concentration of 6 × 10^4^ PFU/μl or approximately 6 × 10^7^ genomic equivalents/μl of extracted RNA. Using gBlock standards, we confirmed the viral copy number to be within 3-fold of the estimated value of 6 × 10^7^ PFU/μl, which is consistent with expected variation from freeze-thaw and qRT-PCR. Quantitative standards were prepared from the WRCEVA sample by serially diluting SARS-CoV-2 RNA (3 × 10^5^, 3 × 10^4^, 3 × 10^3^, 300, 30, and 3 genomic equivalent copies per RT-PCR) and analyzed by SARS-CoV-2 detection assays using the Corman E-gene and CDC N2 primer-probe sets. In parallel, 10-fold serial dilutions of RNA from the SC5688 clinical sample were prepared and tested using the same assays. The standard curve generated from the quantitative standards was used to determine the number of viral genomic equivalents per microliter in the SC5688 sample.

## RESULTS

### Assays using UW RdRp and Corman N-gene primer-probe sets have limits of detection (LODs) of about 790 viral genomic equivalents per reaction.

The first two primer-probes sets that we evaluated were the UW RdRp and the Corman N-gene sets. Assays using these primer-probe sets were used to examine approximately 300 clinical samples of unknown SARS-CoV-2 status. RNA was extracted for these assays using two different systems. The first was the MagNA Pure LC (LC) system, which is able to process 32 samples at a time and elutes RNA into 200 μl of buffer. The second was the MagNA Pure 96 (MP96) system, which is able to process 96 samples at a time and elutes RNA into 100 μl of buffer. One sample (SC5688) out of the 300 was positive for SARS-CoV-2. SC5688 was positive for SARS-CoV-2 regardless of which of the two RNA extraction methods and which of the two primer-probe sets were used.

Sample SC5688 was determined to have a concentration of SARS-CoV-2 genomic equivalents of 3.16 × 10^5^ equivalents/μl. We used sample SC5688 to determine the limit of detection (LOD) of assays using the UW RdRp and Corman N-gene primer-probe sets. For both of these primer-probe sets, 20 out of 20 replicate assays were still positive for SARS-CoV-2 when SC5688 was diluted to 1:2 × 10^3^, the equivalent of about 790 genomic equivalents per reaction. Finally, we tested the specificity of assays using both primer-probe sets by running them on a collection of samples that are positive for respiratory viruses other than SARS-CoV-2. Assays using both sets were found to be 100% specific with no false positives noted in this analysis.

### Assays using the Corman RdRp and E-gene sets were found to have LODs of about 316 viral genomic equivalents per reaction.

We next tested assays using the Corman RdRp and Corman E-gene primer-probe sets. We again used two RNA extraction methods. The LC extraction method was performed using the same protocol as before. However, for the MP96 method, we eluted RNA into 50 μl of buffer instead of 100 μl of buffer to determine whether this would increase the sensitivity of SARS-CoV-2 detection assays. We ran assays using both the Corman RdRp and the Corman E-gene primer-probe sets coupled with both RNA extraction methods on 10 samples confirmed by the WSDOH Public Health Laboratories to be positive for SARS-CoV-2. The results of these tests are shown in Table 1. There was one sample that was positive for assays using the E-gene set when the MP96 system was used but not when the LC system was used, and there were two samples that were positive for assays using the RdRp set when the MP96 system was used but not when the LC system was used. Based on these results, we subsequently used the MP96 RNA extraction system with RNA eluted into 50 μl of buffer for all analyses.

**TABLE 1:** Relative performance of SARS-CoV-2 detection assays using the Corman E-gene and RdRp primer-probe sets and two different RNA extraction methods

Sample ID	Cycle threshold			
	Corman E-gene		Corman RdRp	
	LC^*a*^	MP96^*b*^	LC	MP96
SC5777	27.2	24.9	29.1	29.0
SC5778	33.9	31.9	Negative	34.8
SC5779	37.7	34.7	Negative	36.5
SC5780	16.7	15.1	17.9	19.2
SC5781	17.2	16.2	18.6	20.2
SC5782	24.4	22.6	25.6	26.9
SC5783	18.4	16.9	19.6	20.8
SC5784	Negative	35.4	Negative	Negative
SC5785	32.7	28.9	34.4	32.7
SC5786	27.6	25.6	28.1	29.4

a RNA extraction performed on the MagNA Pure LC system with RNA eluted into 200 μl of buffer.

b RNA extraction performed on the MagNA Pure 96 system with RNA eluted into 50 μl of buffer.

To assess the sensitivity of assays using the Corman RdRp and E-gene primer-probe sets, we ran them on dilutions of sample SC5688. For both of these primer-probe sets, 20 out of 20 replicate assays were still positive for SARS-CoV-2 when SC5688 was diluted to 1:2 × 10^4^, the equivalent of about 316 genomic equivalents per reaction. We also tested the specificities of assays using these sets by running them on our collection of samples positive for various respiratory viruses. Like assays using the UW RdRp and the Corman N-gene primers and probes, those using the Corman RdRp and E-gene sets were 100% specific with no false positives noted.

### Assays using the CDC N1 and N2 primer-probe sets performed better than those using the N3 set.

Given that assays using the Corman RdRp and E-gene primer-probe sets were more sensitive than those using the UW RdRp and Corman N-gene sets, we wanted to compare the former to assays using the primer-probe sets published by the CDC: CDC N1, CDC N2, and CDC N3. We ran assays using these three sets on the 10 positive samples obtained from the WSDOH Public Health Laboratories. The results of these analyses are shown in Table 2. All assays produced positive results for all samples, except for the assay using the CDC N3 primers-probe, which produced a negative result for SC5784. The assay using the Corman RdRp set also produced a negative result for this sample. For the other nine samples, assays using the Corman RdRp set consistently produced the highest cycle thresholds out of all the assays compared in Table 2 followed by assays using the Corman E-gene set.

**TABLE 2:** Relative performance of SARS-CoV-2 detection assays using five different primer-probe sets^*a*^

Sample ID	CDC N1	CDC N2	CDC N3	Corman RdRp	Corman E-gene
SC5777	24.5	23.2	23.3	29.0	24.9
SC5778	30.2	30.6	30.1	34.8	31.9
SC5779	33.3	32.8	32.0	36.5	34.7
SC5780	14.6	13.7	13.9	19.2	15.1
SC5781	15.1	14.1	14.3	20.2	16.2
SC5782	21.8	20.9	21.0	26.9	22.6
SC5783	16.0	14.9	15.6	20.8	16.9
SC5784	36.0	35.6	Negative	Negative	35.4
SC5785	27.8	27.3	27.4	32.7	28.9
SC5786	23.9	24.0	24.3	29.4	25.6

a Cycle thresholds are displayed.

### Assays using the CDC N2 and Corman E-gene primer-probe sets were more sensitive than those using the CDC N1 and Corman RdRp sets and the BGI kit.

Our final analysis was to test the sensitivities and specificities of assays using the CDC primer-probe sets and compare these to the sensitivities and specificities of assays using the Corman RdRp and E-gene sets. Because assays using the N3 set did not perform as well as those using the N1 and N2 sets, we did not include the former set in this analysis. We did, however, include in this analysis an evaluation of the sensitivity and specificity of assays performed using a SARS-CoV-2 test kit from BGI. To directly compare LODs of assays using the Corman RdRp and E-gene sets to those using the N1 and N2 sets and the BGI kit, we ran assays on dilutions ranging from 1:10^4^ to 1:10^7^ of sample SC5688. We again ran 20 duplicate assays with each primer-probe set and with the BGI kit on dilutions of SC5688 as listed in Table 3. The least sensitive assays were the ones that used the Corman RdRp primer-probe set. At a dilution of 1:10^5^ (the equivalent of 63 viral genomic equivalents per reaction), only 17 out of the 20 assays that used this set were positive for SARS-CoV-2. This set of outcomes is significantly different (Fisher’s exact test, *P* value = 7.1 × 10^−3^) from those observed for assays using the Corman E-gene, CDC N1, and CDC N2 primer-probe sets and the BGI kit, for which 20 out of 20 assays were positive. At a dilution of 1:10^6^ (the equivalent of about 6.3 genomic equivalents per reaction), assays using the CDC N2 and the Corman E-gene sets performed equally well (Fisher’s exact test, *P* value = 0.3), with 18 and 17 replicate assays resulting as positive, respectively. The number of replicates that were positive was significantly higher (Fisher’s exact test, *P* value = 2.5 × 10^−6^) for assays using these two primer-probe sets relative to assays using the CDC N1 and Corman RdRP and the BGI kit when assays were run on a 1:10^6^ dilution of sample SC5688.

**TABLE 3:** Relative sensitivities of SARS-CoV-2 detection assays using 4 different primer-probe sets and the BGI testing kit

Primer-probe set or kit	Dilution	Viral copies^*a*^	Sample ID	No. of replicates			% positivity	Mean CT^*b*^^,^^*c*^	SD
				Tested	Positive	Negative			
Corman E-gene	1:10^5^	63	S5	20	20	0	100	33.7	1.13
	1:2 × 10^5^	31.5	S5_2	20	20	0	100	34.7	0.69
	1:5 × 10^5^	12.6	S5_5	20	16	4	80	36.6	1.60
	1:10^6^	6.3	S6	20	17	3	85	37.2	1.34
	1:10^7^	0.63	S7	20	3	17	15	37.9	0.10
Corman RdRp gene	1:10^4^	630	S4	20	20	0	100	35.2	1.34
	1:2 × 10^4^	315	S4_2	20	16	4	80	36.8	1.26
	1:10^5^	63	S5	20	17	3	85	36.6	1.84
	1:10^6^	6.3	S6	20	2	18	10	35.8	1.79
	1:10^7^	0.63	S7	20	1	19	5	37.5	0.00
CDC N1	1:10^5^	63	S5	20	20	0	100	33.7	1.45
	1:2 × 10^5^	31.5	S5_2	20	17	3	85	35.8	1.82
	1:5 × 10^5^	12.6	S5_5	20	12	8	60	36.5	1.58
	1:10^6^	6.3	S6	20	13	7	65	36.2	1.41
	1:10^7^	0.63	S7	20	2	18	10	36.2	0.55
CDC N2	1:10^5^	63	S5	20	20	0	100	33.1	1.26
	1:2 × 10^5^	31.5	S5_2	20	20	0	100	35.1	0.98
	1:5 × 10^5^	12.6	S5_5	20	17	3	85	36.4	1.23
	1:10^6^	6.3	S6	20	18	2	90	36.8	1.35
	1:10^7^	0.63	S7	20	3	17	15	37.2	0.61
BGI kit	1:10^5^	63	S5	20	20	0	100	31.6	2.39
	1:2 × 10^5^	31.5	S5_2	20	20	0	100	33.8	1.31
	1:5 × 10^5^	12.6	S5_5	20	18	2	90	36.2	1.94
	1:10^6^	6.3	S6	20	10	10	50	34.7	2.06
	1:10^7^	0.63	S7	20	0	20	0		

a Number of viral copies (genomic equivalents) per reaction.

b Cycle threshold.

c Only positive results are included in calculation of mean CT.

The specificities of assays using the CDC N1 and N2 primer-probe sets and the BGI kit were also tested using our panel of samples positive for various respiratory viruses. Assays using the N1 and N2 sets and the BGI kit were found to be 100% specific.

## DISCUSSION

Known cases of COVID-19 have now exceeded 375,000 worldwide. While the number of new infections in China appears to be leveling off with fewer identified each day, the number of cases continues to rapidly increase in other nations across the world (3). In the coming days and weeks, many clinical laboratories will be developing and optimizing their own SARS-CoV-2 testing protocols. Maximizing the sensitivity and specificity of these tests is critical to efforts around the world to minimize the impact of this pandemic on global health.

A number of different primer-probe sets for use in SARS-CoV-2 detection assays and SARS-CoV-2 testing kits have been developed and are now available. As we have demonstrated here, the performance characteristics of assays using these primer-probe sets and testing kits are variable. Of the seven different primer-probe sets and one testing kit that we evaluated, all were found to be highly specific with no false-positive results observed when assays were run on samples positive for a number of other respiratory viruses. Variability was, however, observed in the sensitivities of these tests. We found assays using the CDC N2 and Corman E-gene primer-probe sets to be particularly sensitive. Assays using these sets were able to detect SARS-CoV-2 in 10 out of 10 known positive clinical samples. They were also able to reliably detect SARS-CoV-2 in a sample containing only about 6 genomic equivalents of viral RNA. In addition to our evaluation of different assays for SARS-CoV-2 detection, we also show that it is possible to significantly increase capacity for the RNA extraction step of SARS-CoV-2 testing without sacrificing sensitivity.

In summary, we report variable performance characteristics of SARS-CoV-2 detection assays using seven different primer-probe sets and one complete testing kit when applied to clinical samples. While all assays evaluated were highly specific, some, such as those using the CDC N2 and the Corman E-gene sets, were found to be more sensitive than others. These findings will provide important insights on SARS-CoV-2 detection assay design to labs that are currently working to develop their own testing methods. Our results also emphasize the importance of ongoing optimization of viral detection assays following the emergence of novel viral pathogens.

## Supplementary Material

Supplemental file 1
