# Supplementary material for: Comparative Performance of SARS-CoV-2 Detection Assays Using Seven Different Primer-Probe Sets and One Assay Kit
Source: J Clin Microbiol. 2020 May 26;58(6):e00557-20. doi: 10.1128/JCM.00557-20 (PMC7269385; doi:10.1128/JCM.00557-20)
Supplement: Supplemental file 1 [file JCM.00557-20-s0001.pdf]

Supplementary Note 1: Definition of COVID-19 Case

We use the term “case” to denote an individual with laboratory confirmed SARS-CoV-2 infection regardless of clinical symptoms. This aligns with the World Health Organization’s (WHO) definition of a “confirmed case” as “a person with laboratory confirmation of COVID-19 infection, irrespective of clinical signs and symptoms.”(1)

References:

1. World Health Organization. 2020. Global surveillance for COVID-19 caused by human infection with COVID-19 virus. Interim guidance, World Health Organization. https://www.who.int/publications-detail/global-surveillance-for-human-infection-with-novel-coronavirus-(2019-ncov)

Primer / Probe	Sequence (5' to 3')	Target	Number of SARS-CoV2 sequences with mismatches in primer/probe sites* (n=180)
RdRP_SARSr-Forward	GTGARATGGTCATGTGTGGCGG	RdRp (Corman)	
RdRP_SARSr-Reverse	CARATGTTAAASACACTATTAGCATA		
RdRP_SARSr-Probe	***FAM-*** CAGGTGGAACCTCATCAGGAGATGC-***BHQ1***		
N_Sarbeco_Forward	CACATTGGCACCCGCAATC	N-gene (Corman)	1
N_Sarbeco_Reverse	GAGGAACGAGAAGAGGCTTG		
N_Sarbeco_Probe	***FAM-*** ACTTCCTCAAGGAACAACATTGCCA-***BHQ1***		
E_Sarbeco_Forward	ACAGGTACGTTAATAGTTAATAGCGT	E-gene (Corman)	
E_Sarbeco_Reverse	ATATTGCAGCAGTACGCACACA		1
E_Sarbeco_Probe	***FAM-*** ACACTAGCCATCCTTACTGCGCTTCG-***BHQ1***		1
nCoV_2019 Forward	CAAATTCTATGGTGGTTGGCACA	RdRp (UW)	
nCoV_2019 Reverse	GGCATGGCTCTATCACATTTAGG		
CoV_Probe	***FAM-*** ATAATCCCAACCCATRAG-***MGB***		
EXO Forward	GGCGGAAGAACAGCTATTGC	Jellyfish gene (internal control)	N/A
EXO Reverse	GGAACCTAAGACAAGTGTGTTTATGG		N/A
EXO Probe	***VIC-*** AACGCCATCGCACAAT-***MGB***		N/A
CDC N1 Forward	GACCCCAAAATCAGCGAAAT	N-gene (CDC)	1
CDC N1 Reverse	TCTGGTTACTGCCAGTTGAATCTG		
CDC N1 Probe	***FAM-*** ACCCCGCATTACGTTTGGTGGACC-***BHQ1***		1
CDC N2 Forward	TTACAAACATTGGCCGCAAA		
CDC N2 Reverse	GCGCGACATTCCGAAGAA		
CDC N2 Probe	***FAM-*** ACAATTTGCCCCCAGCGCTTCAG-***BHQ1***		1
CDC N3 Forward	GGGAGCCTTGAATACACCAAAA		8
CDC N3 Reverse	TGTAGCACGATTGCAGCATTG		
CDC N3 Probe	***FAM-*** AYCACATTGGCACCCGCAATCCTG-***BHQ1***		1
RNAseP Forward	AGATTTGGACCTGCGAGCG	RNAseP (CDC internal control)	N/A
RNAseP Reverse	GAGCGGCTGTCTCCACAAGT		N/A
RNAseP Probe	***FAM-*** TTCTGACCTGAAGGCTCTGCGCG-***BHQ1***		N/A

Supplementary Table 1: Primer and probe sets used in the current study. A total of 180 SARS-CoV2 sequences (GISAID) were compared to identify polymorphisms in primer/probe sequences.

*Total number of sequences with SNP in primer/probe region. Blank cells represent 100% homology.

N/A: Not applicable. EXO and RNAseP have no homology with SARS-CoV2 sequences.

FAM: 6-carboxyfluorescein

VIC: 2′-chloro-7′phenyl-1,4-dichloro-6-carboxy-fluorescein

BHQ1: Black Hole Quencher-1

MGB: Minor Grove Binder
